# Molecular Survey and Genetic Characterization of *Tetratrichomonas buttreyi* and *Pentatrichomonas hominis* in Horses in Shanxi Province, Northern China

**DOI:** 10.3390/ani16172699

**Published:** 2026-08-31

**Authors:** Dong-Yang Wang, Nan Su, Wen Li, Ze-Dong Zhang, Xun-Zhi Liu, Wen-Wei Gao

**Affiliations:** Shanxi Key Laboratory of Animal Disease Research, Prevention and Control, College of Veterinary Medicine, Shanxi Agricultural University, Jinzhong 030801, China; 17303450339@163.com (D.-Y.W.); sunan1228@163.com (N.S.); 13934936109@163.com (W.L.); zhangzedong0519@126.com (Z.-D.Z.); liuxunzhi0206@126.com (X.-Z.L.)

**Keywords:** *Tetratrichomonas buttreyi*, *Pentatrichomonas hominis*, horses, molecular epidemiology, Shanxi Province

## Abstract

Trichomonads include species of veterinary and zoonotic importance, yet infections with trichomonads in horses remain uncharacterized in Shanxi Province, Northern China. In the present study, we conducted a molecular survey of *Tetratrichomonas buttreyi* and *Pentatrichomonas hominis* using 631 horse fecal samples collected from Datong, Taiyuan, and Yuncheng cities in Shanxi Province between March and June 2023. Nested PCR assays targeting the small subunit ribosomal RNA (SSU rRNA) gene were used to detect the two trichomonad species. The overall molecular prevalences were 3.7% (23/631; 95% CI, 2.5–5.5%) for *T. buttreyi* and 0.8% (5/631; 95% CI, 0.3–1.9%) for *P. hominis*. *T. buttreyi* prevalence differed significantly by region and management mode but not by age or sex. *P. hominis* was detected only in Datong City, resulting in a significant regional difference; however, age, sex, and management mode were not significantly associated with *P. hominis* prevalence. Five representative *T. buttreyi* sequences and one representative *P. hominis* sequence were obtained in the present study. Among these sequences, two *T. buttreyi* sequences shared 100% nucleotide identity with reference sequences from pigs, cattle, and horses; three showed only minor nucleotide variation. The representative *P. hominis* sequence clustered with the zoonotic genotype CC1, suggesting that horses may form part of the broader host network of *P. hominis*, though further work is needed to establish horses’ reservoir competence or confirm their role in the transmission. These findings provide the first molecular evidence of *T. buttreyi* and *P. hominis* in horses from Shanxi Province, and establish a baseline for future surveillance and One Health risk assessments.

## Simple Summary

Trichomonads are anaerobic, single-celled protozoa that commonly colonize the digestive tract of animals. *Pentatrichomonas hominis* is a zoonotic species with increasing veterinary and public health relevance, whereas *Tetratrichomonas buttreyi* is an intestinal trichomonad species whose host range and transmission ecology remain incompletely understood. Meanwhile, no information is available on the epidemiology of the two trichomonad species in horses in China. In the present study, we screened 631 horse fecal samples collected from Shanxi Province for the detection of *P. hominis* and *T. buttreyi* using nested PCR. Overall, *T. buttreyi* (3.7%, 23/631) was more prevalent than *P. hominis* (0.8%, 5/631), and the representative *P. hominis* sequence clustered with the zoonotic CC1 genotype. These results document the infection of *P. hominis* and *T. buttreyi* in horses, provide baseline molecular epidemiological data on two trichomonad species in horses in Shanxi Province, and support the need for continuous surveillance at the interface of horses, other livestock, and humans.

## 1. Introduction

Trichomonads are anaerobic flagellated protozoa within the phylum Parabasalia. Although some species are free-living, most are commensals, endosymbionts, or parasites of vertebrate and invertebrate hosts [1,2]. Intestinal trichomonads have received increasing attention in veterinary parasitology, as several species colonize livestock, companion animals, wildlife, and humans [2]. Such host diversity creates opportunities for fecal–oral transmission in shared environments.

*Tetratrichomonas buttreyi* was first described in pigs and subsequently reported in the bovine digestive tract [3,4]. Its pathogenicity remains debated, although *T. buttreyi* infection has been associated with pathological changes in pigs [5]. Recent molecular surveys in Shanxi Province have detected *T. buttreyi* in pigs, cattle, and donkeys [6,7,8]. Two equine-derived SSU rRNA sequences of *Tetratrichomonas* sp. were deposited into the GenBank database in 2006 [9]; however, molecular epidemiology data of *T. buttreyi* on horses remain unavailable, and the extent to which horses contribute to the maintenance or transmission of *T. buttreyi* is unclear.

*Pentatrichomonas hominis* is an intestinal protozoan with a broad host range. Increasing evidence has expanded the host spectrum to include humans, companion animals, domestic, and wild animals [2,10,11,12,13,14]. Meanwhile, sequences assigned to the genotype CC1 have been detected in humans and multiple animal hosts, with their genetic similarity across host-associated sequences suggesting potential zoonotic transmission [15,16,17]. Although *P. hominis* has been detected among various hosts, this detection cannot distinguish stable host colonization from transient carriage. In addition, a previous study has shown that *P. hominis* can adhere to intestinal epithelial cells, alter cell viability and inflammatory gene expression, and induce macrophage extracellular trap formation through innate immune pathways [18]. Collectively, these findings highlight *P. hominis* as a species with a broad host range, zoonotic concern, and pathogenic potential. Thus, the collection of epidemiological data on *P. hominis* is of great importance.

Because morphology alone cannot reliably distinguish closely related intestinal trichomonads, including *P. hominis* and *T. buttreyi*, PCR-based molecular detection is essential for trichomonad epidemiological investigations [19,20,21]. The SSU rRNA gene is widely used for species detection and sequence comparisons [22], providing prevalence data and genetic information.

Shanxi Province is a major livestock-producing province in Northern China. Horses often share environments with humans and other domestic animals through grazing, husbandry, transport, and recreational activities. Although the presence of *T. buttreyi* and *P. hominis* has been confirmed in other local livestock, the molecular occurrence and role of horses as potential reservoirs of *T. buttreyi* and *P. hominis* remains unknown. To address this gap of knowledge, we investigated the molecular prevalence of *T. buttreyi* and *P. hominis* in horses in Shanxi Province and characterized the obtained SSU rRNA sequences. Also, the risk factors associated with the prevalence of the two trichomonad species and their genetic diversity were analyzed.

## 2. Materials and Methods

### 2.1. Study Sites and Sample Collection

From March to June 2023, 631 fresh horse fecal samples were random collected from three cities in Shanxi Province, Northern China: 269 from Datong City, 261 from Taiyuan City, and 101 from Yuncheng City. Among these, all 261 club horses were sampled in Taiyuan City, whereas 370 pasture-managed horses were sampled in Datong City and Yuncheng City. After spontaneous defecation, approximately 10–20 g of fecal samples was collected from the inner portion of the feces using sterile disposable gloves (to reduce potential contamination from soil and environmental pathogens during collection). For each sample, the animal identification number, location, age, sex, and management were recorded. All samples were placed in insulated boxes with ice packs, and then immediately transported to the Laboratory of Parasitic Diseases, College of Veterinary Medicine, Shanxi Agricultural University, and stored at −20 °C until DNA extraction.

### 2.2. DNA Extraction and PCR Amplification

Genomic DNA was extracted from approximately 200 mg of each fecal sample using the E.Z.N.A.^®^ Stool DNA Kit (Omega Bio-tek Inc., Norcross, GA, USA) according to the manufacturer’s protocol. The extracted DNA was stored at −20 °C until PCR analysis.

To detect *T. buttreyi*, all DNA samples were amplified using a nested PCR assay targeting a 623 bp fragment of the SSU rRNA gene. The primers used in this study are summarized in Table 1. For *T. buttreyi* detection, the first-round PCR conditions were as follows: initial denaturation at 95 °C for 10 min; 30 cycles of denaturation at 95 °C for 30 s, annealing at 60 °C for 1 min, and extension at 72 °C for 1 min; and final extension at 72 °C for 10 min. Following this, the second-round PCR conditions were as follows: initial denaturation at 95 °C for 10 min; 30 cycles of 95 °C for 45 s, 60 °C for 45 s, and 72 °C for 45 s; and final extension at 72 °C for 10 min. Regarding *P. hominis*, nested PCR was performed to amplify a 339 bp fragment of SSU rRNA gene. The PCR amplification procedure was the same as that described above for *T. buttreyi*, with the exception of an annealing temperature of 55 °C for the first-round PCR.

Each PCR was prepared in a final volume of 25 μL containing 2 μL of the genomic DNA template or 2 μL of the first-round PCR product for the second-round reaction, as well as 0.4 μM of each primer, 200 μM of each dNTP, 1.5 mM of MgCl_2_, 2.5 μL of 10× PCR buffer (Mg^2+^ free), and 0.625 U of *Ex*-Taq DNA polymerase (TaKaRa Inc., Dalian, China). Negative controls (deionized water) and positive controls (DNA samples that were previously confirmed to be positive for *T. buttreyi* or *P. hominis*) were included in each PCR round to ensure the accuracy and reliability of the amplification. Second-round PCR products were separated by electrophoresis on 1.5% agarose gels and visualized by ethidium bromide staining.

### 2.3. Sequencing and Phylogenetic Analysis

All 23 *T. buttreyi*-positive and five *P. hominis*-positive PCR products of the expected sizes were gel-purified and subjected to bidirectional Sanger sequencing on a 3730XL automated DNA sequencer (Applied Biosystems by Thermo Fisher Scientific Inc., Waltham, MA, USA) by Sangon Biotech (Shanghai) Co., Ltd. (Shanghai, China). Raw sequences were assembled using Chromas Pro v2.1.3 software. Consensus sequences were compared with reference sequences available in GenBank using BLAST (https://blast.ncbi.nlm.nih.gov/Blast.cgi, accessed on 26 August 2026).

Multiple sequence alignments were generated using representative sequences obtained in this study and closely related GenBank reference sequences. Phylogenetic trees were constructed in MEGA 7, employing the Maximum Likelihood method. Genetic distances were determined using the Kimura two-parameter model. Node support of each branch within the phylogenetic tree was assessed with 1000 replicates as a bootstrap.

### 2.4. Statistical Analysis

Due to the lower or even zero prevalence in certain subgroups, the associations between each parasite’s prevalence and categorical variables were assessed using two-sided Fisher’s test for 2 × 2 tables and Fisher–Freeman–Halton tests for 2 × 3 tables in SPSS 26.0 software (IBM Corp., Chicago, IL, USA). Results were treated as univariable comparisons. Region and management categories were not interpreted as independent factors because they were completely confounded by the sampling structure. A *p*-value < 0.05 was considered statistically significant.

## 3. Results

### 3.1. Prevalence of T. buttreyi and P. hominis in Horses in Shanxi Province

Among the 631 horse fecal samples collected from three cities in Shanxi Province, 23 were positive for *T. buttreyi*, giving an overall prevalence of 3.7% (95% CI, 2.5–5.5%) (Table 2). Regarding region, the highest prevalence was observed in Taiyuan City (8.0%, 21/261; 95% CI, 5.3–12.0%), followed by Datong City (0.7%, 2/269; 95% CI, 0.2–2.7%); no positive samples were detected in Yuncheng City (0/101). The regional difference was significant (*p* < 0.001).

*T. buttreyi* was detected in both age groups, with a prevalence of 3.7% (20/540; 95% CI, 2.4–5.7%) in horses aged ≥ 3 years and 3.3% (3/91; 95% CI, 1.1–9.2%) in horses aged <3 years. No significant age-associated differences were observed (*p* = 1.000). Regarding sex, the prevalence of *T. buttreyi* was 3.8% (4/105; 95% CI, 1.5–9.4%) in intact males, 3.6% (18/494; 95% CI, 2.3–5.7%) in females, and 3.1% (1/32; 95% CI, 0.6–15.7%) in geldings, with no significant differences among the three groups (*p* = 1.000). Regarding management modes, a significantly higher prevalence was observed in horses in club (8.0%, 21/261; 95% CI, 5.3–12.0%) compared to the pasture-managed horses (0.5%, 2/370; 95% CI, 0.1–1.9%) with a significant difference (*p* < 0.001). However, because all club horse samples were collected in Taiyuan City and all pasture horse samples were collected in Datong City or Yuncheng City, the management contrast was inseparable from geographic differences and was not interpreted as an independent management effect.

Five horses were positive for *P. hominis*, giving an overall prevalence of 0.8% (95% CI, 0.3–1.9%) (Table 3). All *P. hominis*-positive samples were detected in Datong City (1.9%, 5/269; 95% CI, 0.8–4.3%), whereas no positive samples were detected in Taiyuan City and Yuncheng City, and the regional difference was statistically significant (*p* = 0.043). *P. hominis* was detected in both age groups (aged ≥3 years: 0.7%, 4/540; aged <3 years: 1.1%, 1/91) and there were no significant differences (*p* = 0.542). Regarding sex, *P. hominis* was detected only in females (1.0%, 5/494), with no significant differences (*p* = 0.686). Regarding management modes, *P. hominis* was detected only in grazing horses (1.4%, 5/370; 95% CI, 0.6–3.1%), and the difference was not statistically significant (*p* = 0.080). Because the city and management categories were completely confounded, neither comparison was interpreted as an independent risk-factor effect.

### 3.2. Genetic Characterization of T. buttreyi Sequences

**Figure 1:**
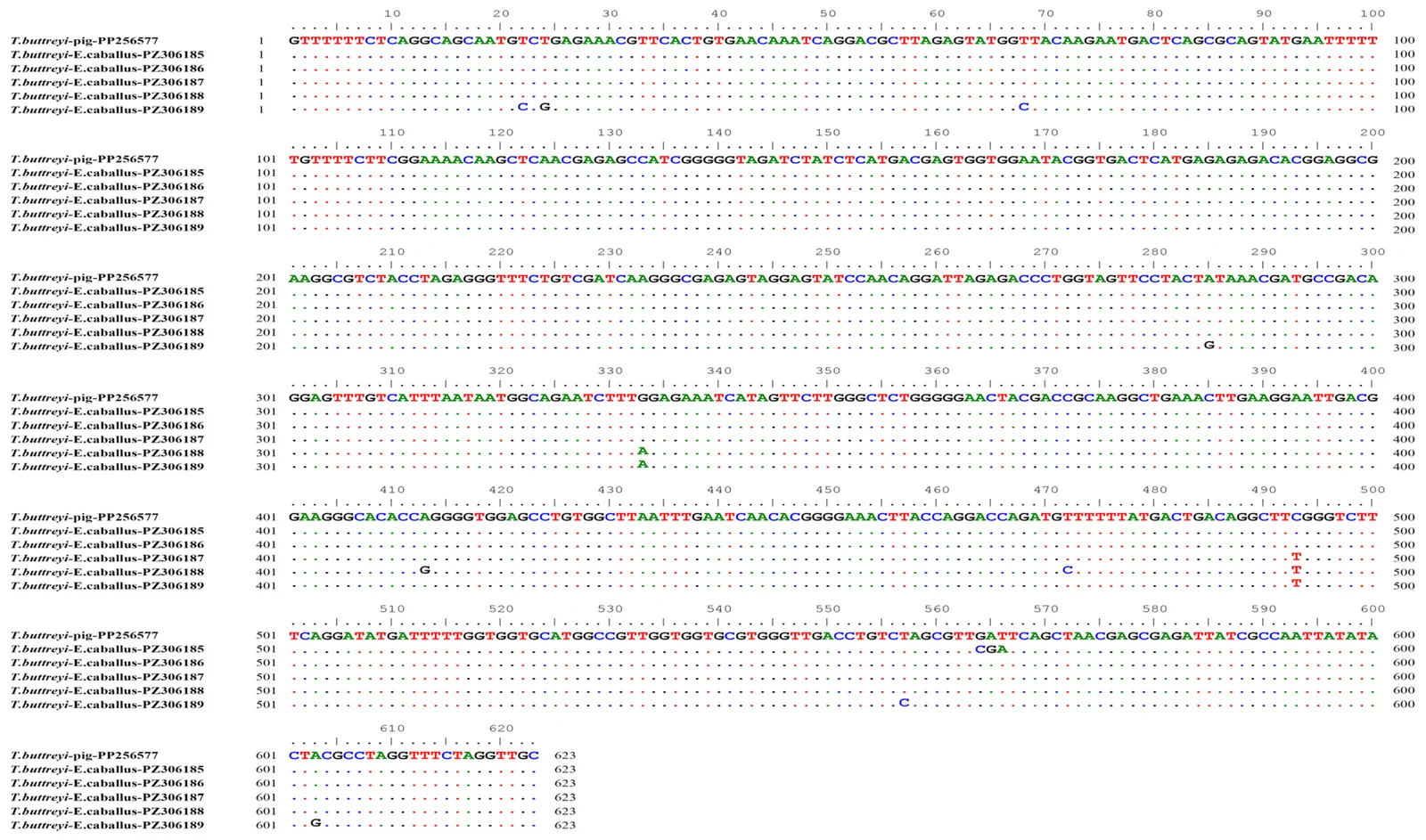
Multiple-sequence alignment of the partial SSU rRNA gene sequences obtained from the horse-derived *T. buttreyi*-positive samples (PZ306185–PZ306189) and the pig-derived *T. buttreyi* reference sequence from China (PP256577). Dots indicate the nucleotide bases identical to the consensus sequence. Colors indicate the four nucleotide bases (A, green; T, red; C, blue; G, black).

After trimming low-quality ends, consensus sequences of a 623 bp SSU rRNA fragment were assembled and aligned within *T. buttreyi*, and sequences sharing 100% identity were assigned to the same variant group. Among 23 *T. buttreyi*-positive samples, five unique haplotypes were obtained by sequence analysis, which were deposited in GenBank under accession numbers PZ306185–PZ306189. As shown in Figure 1, sequences PZ306185 and PZ306186 shared 100% nucleotide identity with the pig-derived *T. buttreyi* reference sequence from China (PP256577). These two sequences were also identical to previously reported sequences from different host species and geographic regions, including a cattle-derived *T. buttreyi* sequence from China (MK880285), an equine-derived sequence from the Czech Republic (AY886859), and pig-derived sequences from Germany (MK801506) and the Philippines (JX565058). The remaining three sequences showed only minor nucleotide variation. Compared with the reference sequences, two to seven single-nucleotide polymorphisms (SNPs) were detected. These sequences shared 99.2–99.8% nucleotide identity with the pig-derived reference sequence PP256577 and the cattle-derived *T. buttreyi* sequence MK880285 from China. No insertions or deletions were detected in any of the five *T. buttreyi* sequences.

**Figure 2:**
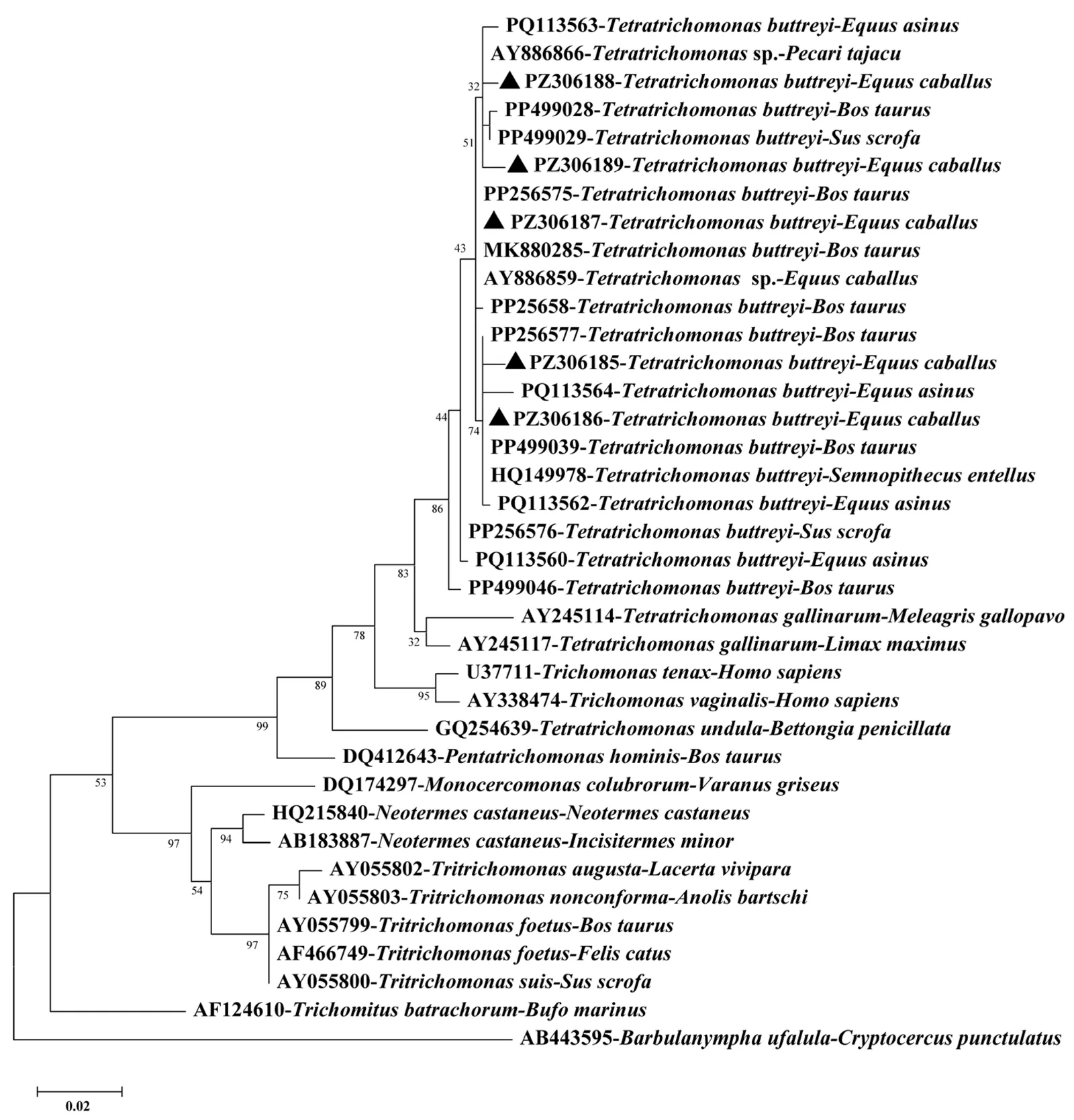
Phylogenetic relationships among trichomonad sequences inferred using the Maximum Likelihood method based on the SSU rRNA nucleotide sequences and the Kimura two-parameter model with Gamma distribution (K2 + G) mode. Black triangles indicate the five representative SSU rRNA sequences of *T. buttreyi* obtained from domestic horses (*Equus caballus*) in this study, with accession numbers PZ306185–PZ306189. AB443595 (*Barbulanympha ufalula*) was used as the outgroup. Numbers at the nodes represent bootstrap support values derived from 1000 replicates; only values above the threshold of 50% are shown. The scale bar represents a genetic distance of 0.02 nucleotide substitutions per site.

Sequence comparisons revealed limited variation among the five representative horse-derived *T*. *buttreyi* sequences obtained in this study (PZ306185–PZ306189). Two sequences were identical to previously deposited *T. buttreyi* sequences in GenBank, whereas the remaining sequences differed by only a small number of nucleotide substitutions. Consistent with these results, phylogenetic analysis placed all five sequences within the *T. buttreyi* clade, which was separated from other trichomonad species with a bootstrap value of 86% (Figure 2).

The horse-derived sequences did not form a distinct host-specific lineage but were interspersed with *T. buttreyi* sequences originating from cattle, pigs, donkeys, primates, and other animal hosts. The previously deposited horse-derived *Tetratrichomonas* sp. sequence AY886859 was also located within the same *T. buttreyi* clade, indicating a close phylogenetic affinity with *T. buttreyi*. The generally short branch lengths within this clade suggest limited genetic divergence in the analyzed sequence region. Nevertheless, because several internal branches were supported by relatively low bootstrap values, the detailed relationships among individual isolates could not be resolved with confidence.

### 3.3. Genetic Characterization of P. hominis Sequences

For *P. hominis*, all five sequences were identical, and one representative was deposited in GenBank under accession number PZ306191. This sequence shared 99.7% nucleotide identity with the fox-derived *P. hominis* reference sequence from China (OM763804). Its core region (101–342 bp) shared 100% nucleotide identity with *P. hominis* sequences from diverse geographic regions and host species, including the Amur tiger (*Panthera tigris* altaica, MZ424463), domestic dog (*Canis lupus* familiaris, KJ404269 and KX136884), raccoon dog (*Nyctereutes procyonoides*, OM763803), donkey (*Equus asinus*, PQ114253), cattle (*Bos taurus*, DQ412643), goat (*Capra hircus*, JX565028), and human (*Homo sapiens*, AF124609). Only three single-nucleotide variants were detected within positions 1–100. No insertions or deletions were identified. Phylogenetic analysis showed that the equine-derived *P. hominis* sequence clustered closely with the zoonotic genotype CC1 sequences reported from human clinical samples and diverse animal hosts.

**Figure 3:**
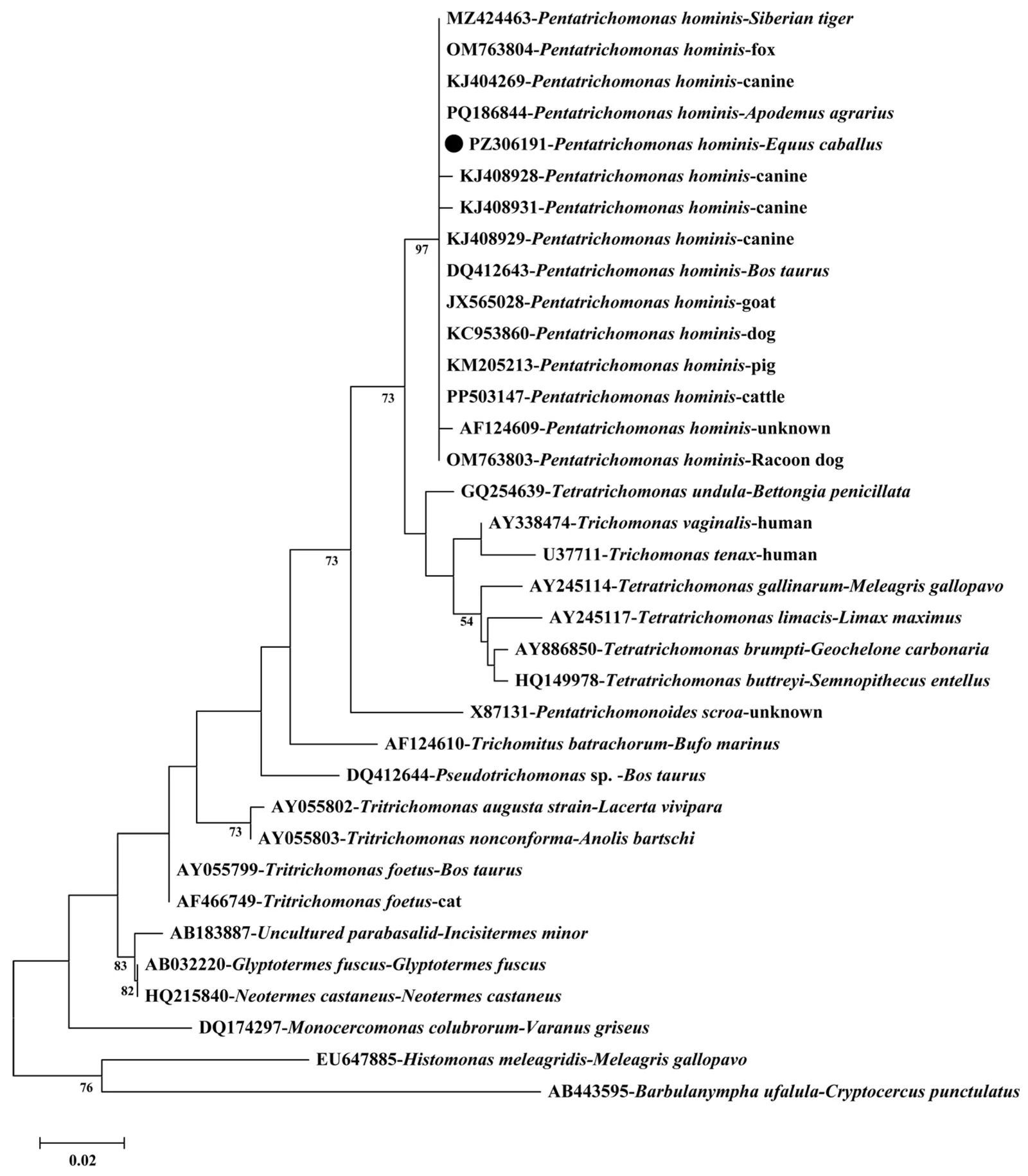
Phylogenetic relationships among trichomonad sequences inferred using the Maximum Likelihood method based on SSU rRNA nucleotide sequences and the Kimura two-parameter model with Gamma distribution (K2 + G) mode. The black circle indicates the representative SSU rRNA sequence of *P. hominis* obtained from a domestic horse (*Equus caballus*) in this study, with accession number PZ306191. AB443595 (*Barbulanympha ufalula*) was used as the outgroup. Numbers at the nodes represent bootstrap support values derived from 1000 replicates; only values above the threshold of 50% are shown. The scale bar represents a genetic distance of 0.02 nucleotide substitutions per site.

A sequence comparison showed that the horse-derived *P*. *hominis* sequence obtained in this study (PZ306191) exhibited a high sequence identity with previously characterized genotype CC1 sequences. Consistent with the sequence comparison, phylogenetic analysis placed PZ306191 within a well-supported monophyletic *P. hominis* clade (bootstrap value = 97%) containing reference sequences previously assigned to genotype CC1 (Figure 3). This clade included isolates originating from a wide range of animal hosts. PZ306191 was interspersed among these reference sequences and did not form a distinct horse-associated lineage. The *P. hominis* clade was clearly separated from other trichomonad taxa included in the analysis. Together, the high sequence similarity and phylogenetic placement support the identification of PZ306191 as *P. hominis* genotype CC1.

## 4. Discussion

This study provides molecular evidence that *T. buttreyi* and *P. hominis* occur in horses in Shanxi Province, China. Given that microscopy cannot reliably distinguish morphologically similar trichomonads, SSU rRNA-based molecular detection has been widely used for species identification in epidemiological surveys [6,7,22]. Thus, in the present study, we performed a prevalence assessment and genetic analysis of *T. buttreyi* and *P. hominis* in horses in Shanxi Province based on a nested PCR amplification of a fragment of the SSU rRNA gene combined with sequence analysis.

Our study showed that the overall prevalence of *T. buttreyi* in horses was 3.7% (23/631), lower than that previously reported in donkeys (25.4%), cattle (74.5%) and pigs (49.2%) from Shanxi Province [6,7,8]. Differences between studies may be due to host species, husbandry, sampling season, environmental exposure, and local management. Regarding risk factors, no significant associations were observed between *T. buttreyi* prevalence and host age or sex in exact univariable comparisons in this study, consistent with previous reports on other intestinal trichomonas species [23]. Instead, environmental variables, such as feeding density and fecal management practices, are considered to be the primary drivers of trichomonad transmission [24]. A previous study has demonstrated that the infection dynamics of trichomonad protozoa vary significantly under different husbandry and ecological conditions [9]. Although a higher prevalence was observed in the sampled club horses than in the pasture horses, all club horses came from Taiyuan City and all pasture horses from Datong or Yuncheng Cities in the present study. The observed contrast therefore cannot be separated into independent city and management effects. Contact density, shared equipment, housing, fecal-removal practices, and feed or water hygiene are plausible hypotheses, but these variables were not assessed. More studies that sample both management systems within the same regions are required in the future to evaluate their correlation.

The partial SSU rRNA sequences of *T. buttreyi* obtained from horses in this study showed limited nucleotide variation. Two sequences were identical to corresponding sequences from pigs and cattle in other regions [6,7], whereas the remaining sequences differed by only a few SNPs. This observation may support the broad host range of *Tetratrichomonas* species [9], and was consistent with the recent findings in donkeys and Milu deer [8,25]. However, identical or similar SSU rRNA sequences cannot verify direct transmission among host species. The SSU rRNA locus has insufficient resolution for fine-scale transmission inference. Therefore, validated multilocus sequence typing, whole-genome sequencing, or animal infection experiments will be needed to clarify whether identical sequences reflect horizontal transmission and host specificity of these parasites [26,27].

Notably, the overall prevalence of *P. hominis* in horses in the present study was extremely low (0.8%, 5/631), and all positive samples were from Datong City. Similarly, a comparable prevalence of *P. hominis* has recently been reported in donkeys, with an overall prevalence of 0.7% [8]. The geographic locations may reflect localized exposure, but the small number of positive samples limits inference, and the complete overlap between the sampling region and management prevents interpretation of either variable as an independent factor. A similar low prevalence has been reported in some wildlife surveys, whereas higher prevalences have been found in other animal populations, including companion animals, pigs, farmed foxes, raccoon dogs, and cattle [6,10,15,28,29]. Together, these studies indicate that the prevalence of *P. hominis* varies substantially by host species, region, sampling strategy, and ecological setting.

Five *P. hominis* sequences obtained in this study were clustered closely with the genotype CC1, which has been reported from humans and multiple animal hosts [10,15,16,30,31]. Previous studies indicated that all *P. hominis*-positive fecal samples from donkeys were also clustered in the genotype CC1 in Shanxi Province, and *P. hominis* has also been detected in dogs and individual boa and owl specimens in the Philippines [8,32]. These findings extend the host range, but a single fecal- or specimen-based PCR results cannot distinguish stable colonization from transient passage or contamination. Previous studies have shown that *P. hominis* can adhere to intestinal epithelial cells, alter cell morphology and viability, and induce inflammatory responses, indicating the pathogenic potential of *P. hominis* [18,33]. However, as standard systematic clinical examinations were not performed in this study, no obvious clinical symptoms were observed in all sampled horses during collection of fecal samples, indicating that the pathogenic potential of *P. hominis* in horses remains unknown.

This study has several limitations. First, sampling was confined to three cities and a short period of time, precluding the assessment of annual and seasonal patterns. Second, the sampling region and management categories were completely confounded, preventing an estimation of their independent effects. Finally, the analysis relied on SSU rRNA sequences, which are suitable for molecular detection but may not resolve fine-scale transmission patterns. Despite these limitations, the present study has provided valuable baseline data for horse trichomonads in Shanxi Province. Future work should expand sampling across different seasons, and combine multilocus markers or a whole-genome sequencing approach with clinical observations to clarify whether horses contribute meaningfully to regional transmission networks, as well as whether *P. hominis* in horse populations has practical implications for animal health or public health risks.

## 5. Conclusions

This study provides the first molecular evidence of *T. buttreyi* and *P. hominis* infection in horses in Shanxi Province, Northern China, with both species showing low overall prevalence and thus, documenting the occurrence of *T. buttreyi* and *P. hominis* in horses in Shanxi Province, China. The *T. buttreyi* sequences obtained in the present study were highly similar or identical to sequences from other domestic animals, indicating limited genetic divergence. Furthermore, the representative *P. hominis* sequence obtained in the present study was clustered closely with the genotype CC1, suggesting a potential role of horses in the regional transmission network. These findings provide baseline epidemiological data and highlight the need for more extensive future sampling, higher-resolution molecular typing, and clinical observations to better understand the transmission routes, host specificity, and the potential One Health significance of equine trichomonads.

## Figures and Tables

**Table 1:** Primers used in this study.

Species	Target Gene	Primers ID	Sequences (5′ to 3′)	Annealing Temperatures (°C)	Fragment Length (bp)	Reference
*T. buttreyi*	SSU rRNA	F1	GCGCCTGAGAGATAGCGACTA	60		[8,21]
		R1	GGACCTGTTATTGCTACCCTCTTC			
		bF2	GTTTTTTCTCAGGCAGCAATG	60		
		bR2	GCAACCTAGAAACCTAGGCG		623 bp	
*P. hominis*	SSU rRNA	Ph1	ATGGCGAGTGGTGGAATA	59		[8,21]
		Ph2	CCCAACTACGCTAAGGATT			
		Th3	TGTAAACGATGCCGACAGAG	61	339 bp	
		Th5	CAACACTGAAGCCAATGCGAGC			

**Table 2:** Prevalence of *Tetratrichomonas buttreyi* and associated factors in horses in Shanxi Province, Northern China.

Factor	Category	No. Examined	No. Positive	Prevalence % (95% CI)	p-Value
Region	Datong	269	2	0.7 (0.2–2.7)	<0.001
	Taiyuan	261	21	8.0 (5.3–12.0)	
	Yuncheng	101	0	0.0 (0.0–3.7)	
Age	≥3 years	540	20	3.7 (2.4–5.7)	1.000
	<3 years	91	3	3.3 (1.1–9.2)	
Sex	Male	105	4	3.8 (1.5–9.4)	1.000
	Female	494	18	3.6 (2.3–5.7)	
	Gelding	32	1	3.1 (0.6–15.7)	
Management	Club	261	21	8.0 (5.3–12.0)	<0.001
	Pasture	370	2	0.5 (0.1–1.9)	
Total	-	631	23	3.7 (2.5–5.5)	-

**Table 3:** Prevalence of *Pentatrichomonas hominis* and associated factors in horses in Shanxi Province, Northern China.

Factor	Category	No. Examined	No. Positive	Prevalence % (95% CI)	p-Value
Region	Datong	269	5	1.9 (0.8–4.3)	0.043
	Taiyuan	261	0	0.0 (0.0–1.5)	
	Yuncheng	101	0	0.0 (0.0–3.7)	
Age	≥3 years	540	4	0.7 (0.3–1.9)	0.542
	<3 years	91	1	1.1 (0.2–6.0)	
Sex	Male	105	0	0.0 (0.0–3.5)	0.686
	Female	494	5	1.0 (0.4–2.3)	
	Gelding	32	0	0.0 (0.0–10.7)	
Management	Club	261	0	0.0 (0.0–1.5)	0.080
	Pasture	370	5	1.4 (0.6–3.1)	
Total	-	631	5	0.8 (0.3–1.9)	-

## Data Availability

The datasets supporting the results of this article have been submitted to GenBank, and the analytical parameters required to reproduce the sequence alignments and phylogenetic analyses are included in the article.

## References

[1] Adl S.M., Simpson A.G., Lane C.E., Lukes J., Bass D., Bowser S.S., Brown M.W., Burki F., Dunthorn M., Hampl V. (2012). The revised classification of eukaryotes. J. Eukaryot. Microbiol..

[2] Maritz J.M., Land K.M., Carlton J.M., Hirt R.P. (2014). What is the importance of zoonotic trichomonads for human health?. Trends Parasitol..

[3] Jensen E.A., Hammond D.M. (1964). A morphological study of Trichomonads and related flagellates from the bovine digestive tract. J. Protozool..

[4] Castella J., Munoz E., Ferrer D., Gutierrez J.F. (1997). Isolation of the trichomonad *Tetratrichomonas buttreyi* (Hibler et al., 1960) Honigberg, 1963 in bovine diarrhoeic faeces. Vet. Parasitol..

[5] Mostegl M.M., Richter B., Nedorost N., Maderner A., Dinhopl N., Weissenbock H. (2011). Investigations on the prevalence and potential pathogenicity of intestinal trichomonads in pigs using in situ hybridization. Vet. Parasitol..

[6] Wang Y.X., Jia T., Wang Z.R., Wang J.L., Zhang Z.D., Wu Z.X., Gao W.W., Zhu X.Q., Liu Q. (2025). Molecular identification and survey of *Tetratrichomonas buttreyi* and *Pentatrichomonas hominis* in cattle in Shanxi province, north China. Animals.

[7] Wang Z.R., Fan Q.X., Wang J.L., Zhang S., Wang Y.X., Zhang Z.D., Gao W.W., Zhu X.Q., Liu Q. (2024). Molecular identification and survey of Trichomonad species in pigs in Shanxi province, north China. Vet. Sci..

[8] Xiao H.D., Zhang S., Lv Y.H., Zhang Z.D., Su N., Li L.L., Zhu X.Q., Xie S.C., Gao W.W. (2024). First molecular detection and genetic characterization of *Tetratrichomonas buttreyi* and *Pentatrichomonas hominis* in donkeys in Shanxi province, China. Animals.

[9] Cepicka I., Hampl V., Kulda J., Flegr J. (2006). New evolutionary lineages, unexpected diversity, and host specificity in the parabasalid genus *Tetratrichomonas*. Mol. Phylogenet Evol..

[10] Tang Y., Wang H.T., Li J.H., Hou Q.Y., Qin S.Y., Ma H., Qin Y., Zhao Q., Elsheikha H.M., Liu S. (2025). Prevalence of *Pentatrichomonas hominis* infection in wild rodents. BMC Vet. Res..

[11] Kamaruddin M., Tokoro M., Rahman M.M., Arayama S., Hidayati A.P., Syafruddin D., Asih P.B., Yoshikawa H., Kawahara E. (2014). Molecular characterization of various trichomonad species isolated from humans and related mammals in Indonesia. Korean J. Parasitol..

[12] Bastos B.F., Brener B., de Figueiredo M.A., Leles D., Mendes-de-Almeida F. (2018). Pentatrichomonas hominis infection in two domestic cats with chronic diarrhea. JFMS Open Rep..

[13] Compaore C., Kemta Lekpa F., Nebie L., Niamba P., Niakara A. (2013). *Pentatrichomonas hominis* infection in rheumatoid arthritis treated with adalimumab. Rheumatology.

[14] Zhang N., Zhang H., Yu Y., Gong P., Li J., Li Z., Li T., Cong Z., Tian C., Liu X. (2019). High prevalence of *Pentatrichomonas hominis* infection in gastrointestinal cancer patients. Parasit. Vectors.

[15] Hou Q.Y., Wang H.T., Lu X.L., Jiang J., Wang Y.F., Bao G.R., Qin Y., Liu S., Li L., Wang Y.Y. (2026). Prevalence and genotype of *Pentatrichomonas hominis* in farmed Arctic foxes (*Vulpes lagopus*) in Northern China. Vector Borne Zoonotic Dis..

[16] Lu N., Wang H.T., Hou Q.Y., Qin Y., Li X.M., Yang X., Jiang J., Liu S. (2026). Epidemiology of *Pentatrichomonas hominis* in farmed mink and raccoon dogs across five provinces in China. Foodborne Pathog. Dis..

[17] Li W.C., Wang K., Gu Y. (2018). Occurrence of *Blastocystis* sp. and *Pentatrichomonas hominis* in sheep and goats in China. Parasit. Vectors.

[18] Zhu Y., Cai H., Fang S., Shen H., Yan Z., Wang D., Qi N., Li J., Lv M., Lin X. (2024). Unraveling the pathogenic potential of the *Pentatrichomonas hominis* PHGD strain: Impact on IPEC-J2 cell growth, adhesion, and gene expression. Parasite.

[19] Kim Y.A., Kim H.Y., Cho S.H., Cheun H.I., Yu J.R., Lee S.E. (2010). PCR detection and molecular characterization of *Pentatrichomonas hominis* from feces of dogs with diarrhea in the Republic of Korea. Korean J. Parasitol..

[20] Mostegl M.M., Richter B., Nedorost N., Maderner A., Dinhopl N., Kulda J., Liebhart D., Hess M., Weissenbock H. (2010). Design and validation of an oligonucleotide probe for the detection of protozoa from the order Trichomonadida using chromogenic in situ hybridization. Vet. Parasitol..

[21] Gookin J.L., Stauffer S.H., Levy M.G. (2007). Identification of *Pentatrichomonas hominis* in feline fecal samples by polymerase chain reaction assay. Vet. Parasitol..

[22] Ceza V., Kotyk M., Kubankova A., Yubuki N., Stahlavsky F., Silberman J.D., Cepicka I. (2022). Free-living Trichomonads are Unexpectedly Diverse. Protist.

[23] Gookin J.L., Stebbins M.E., Hunt E., Burlone K., Fulton M., Hochel R., Talaat M., Poore M., Levy M.G. (2004). Prevalence of and risk factors for feline *Tritrichomonas foetus* and *Giardia* infection. J. Clin. Microbiol..

[24] Alves B.S.G., Bacon A., Langridge K., Papasouliotis K., Handel I., Anderson N.E., Meredith A.L. (2023). Epidemiology of a hybrid swarm: Evidence of 11 feline infectious agents circulating in a population of sympatric European wildcat hybrids and free-living domestic cats, in Scotland. Transbound. Emerg. Dis..

[25] Zhang Y., Li Z., Song X., Xiao G., He L., Bai J., Zhong Z., Tian L., Chang Y., Li Z. (2025). Genetic diversity of Trichomonads from Milu deer (*Elaphurus davidianus*) in China. Parasite.

[26] Arabkhazaeli F., Madani S.A., Ghorbani A. (2020). Parasitological and molecular survey of scattered parasitism by trichomonads in some avian species in Iran. Avian Pathol..

[27] Pedraza-Diaz S., Arranz-Solis D., Gomez-Couso H., Fuschs L., Fort M., Rengifo-Herrera C., Navarro-Lozano V., Ortega-Mora L.M., Collantes-Fernandez E. (2019). Multilocus analysis reveals further genetic differences between *Tritrichomonas foetus* from cats and cattle. Vet. Parasitol..

[28] Zou Y., Yao Z.W., Xiao T., Ma Y.R., He J., Chen L.M., Chen X.Q., Chen N. (2025). Emerging trichomonad infections in companion animals: Rapid visual detection of *Pentatrichomonas hominis* and *Tritrichomonas foetus* using an RPA-CRISPR/Cas12a assay. Transbound. Emerg. Dis..

[29] Lu P., Zhu Y., Cai H., Shen H., Fang S., Wang D., Yan Z., Liao S., Qi N., Lv M. (2025). Prevalence and genetic diversity of *Pentatrichomonas hominis* in pig populations in Guangdong and Anhui Provinces, China. Parasite.

[30] Li W.C., Ying M., Gong P.T., Li J.H., Yang J., Li H., Zhang X.C. (2016). *Pentatrichomonas hominis*: Prevalence and molecular characterization in humans, dogs, and monkeys in Northern China. Parasitol. Res..

[31] Liu S., Li J.H., Qin S.Y., Jiang J., Wang Z.J., Ma T., Zhu J.H., Geng H.L., Yan W.L., Xue N.Y. (2025). Existence of *Pentatrichomonas hominis* in Tibetan Antelope (*Pantholops hodgsonii*). Front. Vet. Sci..

[32] Dimasuay K.G., Rivera W.L. (2013). Molecular characterization of trichomonads isolated from animal hosts in the Philippines. Vet. Parasitol..

[33] Zhai Q.A., Zhang X.C., Zhang H.B., Li J.H., Gong P.T., Wang X.C., Li X., Zhang X., Zhang N. (2025). *Pentatrichomonas hominis* induces extracellular traps formation of macrophages via the TLR2/NADPH/PAD4 pathway. Parasit. Vectors.

